# Chemical Evolution of Rhinovirus Identifies Capsid-Destabilizing Mutations Driving Low-pH-Independent Genome Uncoating

**DOI:** 10.1128/JVI.01060-21

**Published:** 2022-01-26

**Authors:** Luca Murer, Anthony Petkidis, Thomas Vallet, Marco Vignuzzi, Urs F. Greber

**Affiliations:** a Department of Molecular Life Sciences, University of Zurich, Zurich, Switzerland; b Institut Pasteur, Viral Populations and Pathogenesis Unit, Department of Virology, CNRS UMR 3569, Paris, France; Hudson Institute of Medical Research

**Keywords:** ammonium chloride, bafilomycin A1, capsid stability, host-targeting drugs, low-pH endosome, neutral pH, niclosamide, picornavirus reverse genetics, viral protein VP1, VP3, virus entry and uncoating

## Abstract

Rhinoviruses (RVs) cause recurrent infections of the nasal and pulmonary tracts, life-threatening conditions in chronic respiratory illness patients, predisposition of children to asthmatic exacerbation, and large economic cost. RVs are difficult to treat. They rapidly evolve resistance and are genetically diverse. Here, we provide insight into RV drug resistance mechanisms against chemical compounds neutralizing low pH in endolysosomes. Serial passaging of RV-A16 in the presence of the vacuolar proton ATPase inhibitor bafilomycin A1 (BafA1) or the endolysosomotropic agent ammonium chloride (NH_4_Cl) promoted the emergence of resistant virus populations. We found two reproducible point mutations in viral proteins 1 and 3 (VP1 and VP3), A2526G (serine 66 to asparagine [S66N]), and G2274U (cysteine 220 to phenylalanine [C220F]), respectively. Both mutations conferred cross-resistance to BafA1, NH_4_Cl, and the protonophore niclosamide, as identified by massive parallel sequencing and reverse genetics, but not the double mutation, which we could not rescue. Both VP1-S66 and VP3-C220 locate at the interprotomeric face, and their mutations increase the sensitivity of virions to low pH, elevated temperature, and soluble intercellular adhesion molecule 1 receptor. These results indicate that the ability of RV to uncoat at low endosomal pH confers virion resistance to extracellular stress. The data endorse endosomal acidification inhibitors as a viable strategy against RVs, especially if inhibitors are directly applied to the airways.

**IMPORTANCE** Rhinoviruses (RVs) are the predominant agents causing the common cold. Anti-RV drugs and vaccines are not available, largely due to rapid evolutionary adaptation of RVs giving rise to resistant mutants and an immense diversity of antigens in more than 160 different RV types. In this study, we obtained insight into the cell biology of RVs by harnessing the ability of RVs to evolve resistance against host-targeting small chemical compounds neutralizing endosomal pH, an important cue for uncoating of normal RVs. We show that RVs grown in cells treated with inhibitors of endolysosomal acidification evolved capsid mutations yielding reduced virion stability against elevated temperature, low pH, and incubation with recombinant soluble receptor fragments. This fitness cost makes it unlikely that RV mutants adapted to neutral pH become prevalent in nature. The data support the concept of host-directed drug development against respiratory viruses in general, notably at low risk of gain-of-function mutations.

## INTRODUCTION

Rhinoviruses (RVs) cause a majority of the common cold incidences worldwide (1). In the United States alone, lost workdays were estimated to be dozens of millions per year, with almost $3 billion (U.S. dollars [USD]) of over-the-counter remedy cost and an annual economic burden of 40 billion USD (2, 3). RV infections affect the upper and lower respiratory tracts and have been associated with severe disease course in patients with chronic obstructive pulmonary disease, asthma, and cystic fibrosis (4).

RVs belong to the genus *Enterovirus* of the *Picornaviridae* family, comprise more than 160 types, and are grouped into three species, A, B, and C (5). Minor group RV-A members use the low-density lipoprotein receptor (LDLR) for attachment and internalization, while major group RV-A and RV-B use intercellular adhesion molecule 1 (ICAM-1), and RV-C uses cadherin-related family member 3 (CDHR3) (6, 7). LDLR binds to minor group RVs on the ring-shaped mesa around the 5-fold axis, whereas ICAM-1 penetrates into the canyon, reaching into the hydrophobic pocket at the floor of the canyon, where a lipophilic pocket factor is located (8, 9). Upon ICAM-1 binding or incubation of virus with desaturated albumin, the pocket factor is released and gives rise to a metastable virion conformation, the activated particle (A-particle). This transition can be blocked by stabilizing compounds binding to the capsid pocket, for example, the WIN (Sterling-Winthrop) compound pleconaril, a broad A- and B-type RV inhibitor (10–13). The A-particle has shed the internal VP4 and exposed the N termini of VP1 proteins (60 per virion) to the outside of the capsid, as shown with coxsackie A virus (14). The VP1 N terminus is hydrophobic and interlocks with host cell membranes to tether the particle, as shown with poliovirus (15, 16). The RNA genome either is ejected from the capsid through an opening at the 2-fold axis or escapes by pentamer disassembly, as shown with enterovirus 71A or echovirus 18 (EV-18), respectively (17, 18). The transition from native to metastable A-particles is impeded by a large energy barrier, the so-called enthalpy of activation (19). For example, receptor binding or low-pH exposure of RV-B14 lowers this barrier (20–22). Conversely, binding of capsid-stabilizing agents increases the energy barrier and stiffens the capsid (19, 23). This coincides with decreased particle breathing and reduced externalization of VP4 and VP1 N termini (24). Intriguingly, repeated passage of low-pH-exposed RV-B14 gives rise to point mutations in VP1 that render the virus low-pH resistant, showing that native virus particles can evolve to increase stability (25).

Virus evolution can be measured in real time, as virus replication is fast and error-prone. RNA viruses with small genomes and polymerases lacking proofreading functions, such as RVs, undergo one mutation per genome per replication (26–29). This generates diversity and variants that rapidly adapt to changing environments. Intriguingly, viruses with increased fidelity of RNA-dependent RNA polymerases (RdRPs) have a fitness disadvantage compared to their natural variants, and those with overly imprecise RdRPs are at risk of lethal mutagenesis (30, 31). RV evolution and the large variability of RVs, with more than 160 types across three species, make it difficult to develop antiviral drugs and vaccines (5; reviewed in references 32, 33, and 34).

Host-targeting antivirals are a viable alternative to direct virus-targeting compounds and have significant advantages, especially if signaling or metabolic functions critical for the virus are altered without compromising the host (35–43). In this study, we investigated the importance of acidic endosomal pH in virus entry and uncoating. Many animal viruses, including RVs, use low endosomal pH to induce conformational changes in their envelope or capsid proteins to trigger the fusion of the lipid envelope with the limiting endosomal membrane or reduce the stability of the capsid and thereby enhance genome uncoating (44–46). We mapped the evolution of RV-A16 in cells treated with different inhibitors of endolysosomal acidification. The virus was made pH independent by lowering its energy barrier for A-particle formation and introducing destabilizing point mutations at the interface between protomers, the building blocks of the capsid made up of one VP1, VP2, VP3, and VP4. The pH-independent virions were more labile than the parental particles and spontaneously underwent uncoating after receptor binding, temperature increase, or exposure to ionic conditions mimicking the endosomal milieu at neutral pH. The results show that RV-A16 compensates for the absence of the low-pH uncoating cue in endosomes by lowering the stability of its capsid at an overall fitness cost compared to the case with wild-type (wt) RV-A16.

## RESULTS

### Inhibition of endolysosomal acidification selects for RV-A16 cross resistant to different acidification inhibitors.

**FIG 1:**
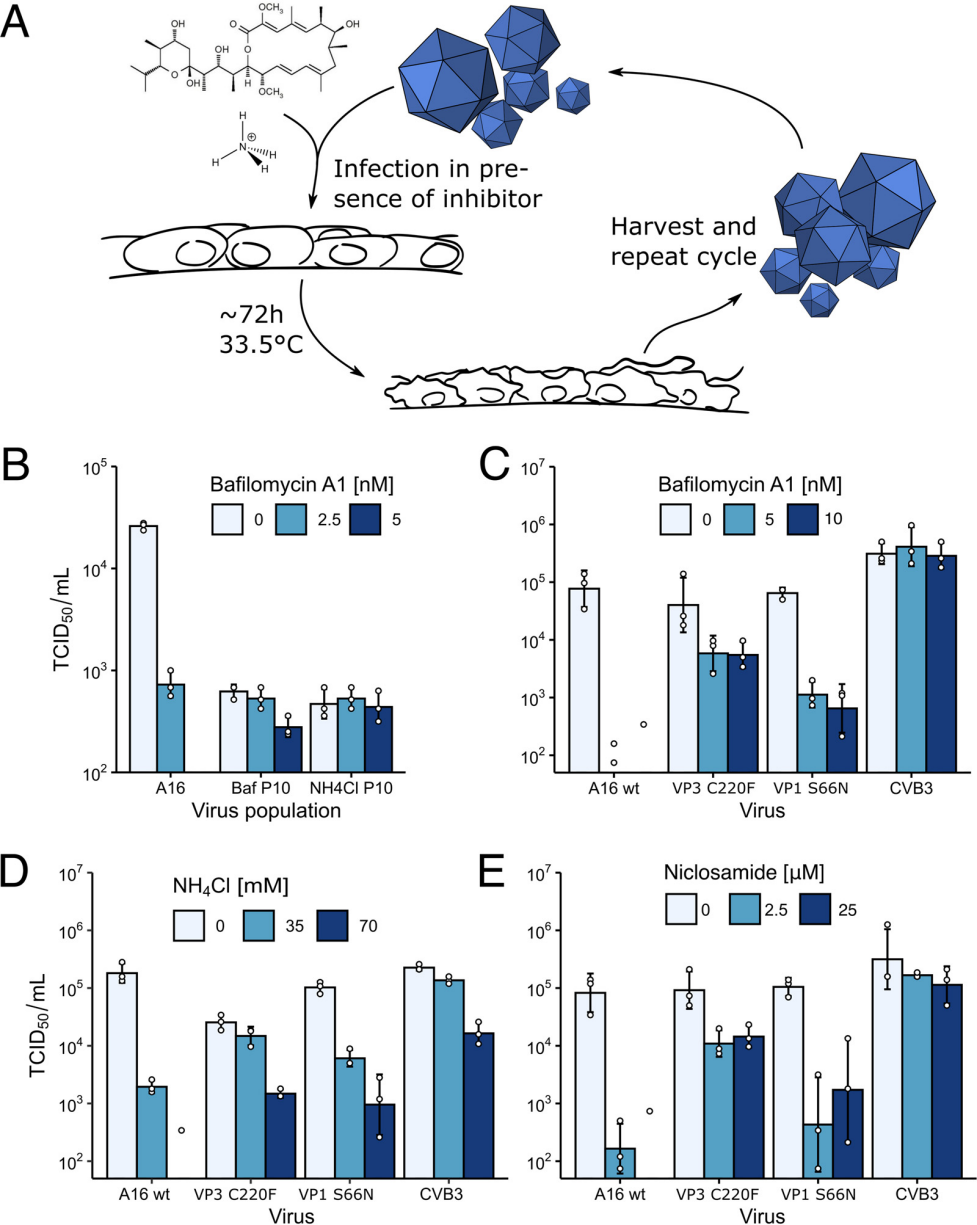
RVs evolve resistance against endolysosomal acidification inhibitors. (A) Schematic depiction of the RV-A16 selection scheme. HeLa-Ohio cells were infected at an MOI of 0.01 with RV-A16 at 33.5°C in the presence of either BafA1 or NH_4_Cl and incubated until ∼90% cytopathic effect occurred. A small volume of the supernatant was transferred to fresh cells with increased inhibitor concentrations. (B) BafA1- and NH_4_Cl-resistant RV-A16 after 10 passages. The harvested populations were resistant against BafA1 or NH_4_Cl after passage 10. Resistant populations were sequenced, and identified mutations affecting structural proteins were introduced into an infectious clone of RV-A16. (C to E) Cross-resistance of RV-A16 mutants. Both the G2274U (VP3-C220F; obtained by passaging in BafA1) and the A2526G (VP1-S66N; passaging in NH_4_Cl) mutants gave rise to cross-resistance against BafA1, NH_4_Cl, and niclosamide, whereby the G2274U mutant was more resistant against the compounds than the A2526G mutant.

**FIG 2:**
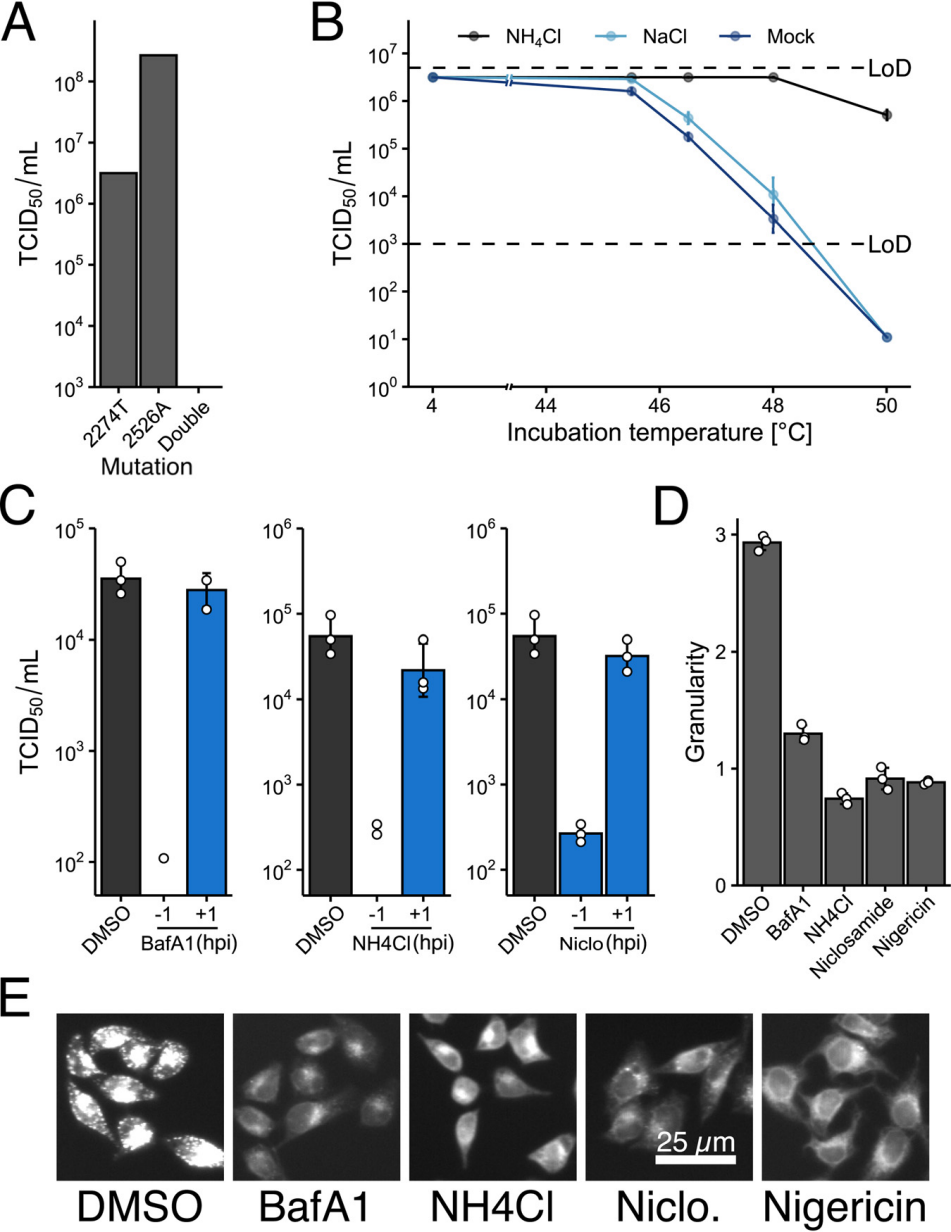
The G2274U/A2526G double mutant is not viable, NH_4_Cl stabilizes wt RV-A16, and endosomal acidification inhibitors act predominantly on virus entry. (A) Formation of infectious units from transfected single but not the double mutant genomic RNAs. *In vitro*-transcribed RNA carrying the indicated mutations was transfected into HeLa-Ohio cells. Newly generated virus was harvested and titers were determined. The observed titer for the double mutant virus preparation was below the limit of detection in the TCID_50_ assay, while the single mutants reached titers above 10^6^ TCID_50_ units per mL. Data from one representative experiment are shown. (B) NH_4_Cl protects RV-A16 from heat inactivation. RV-A16 was incubated at different temperatures in DMEM containing 2% FCS for 20 min, with or without 50 mM NH_4_Cl or 50 mM NaCl. The presence of NH_4_Cl markedly protected RV-A16 from losing infectivity up to about 50°C, whereas under the control conditions, RV-A16 rapidly lost infectivity above 46°C. The dashed line indicates the limit of detection (LoD). (C) BafA1, NH_4_Cl, and niclosamide inhibit RV-A16 infection if added before but not after virus entry. HeLa-Ohio cells were treated with inhibitors at the indicated time and infected with wt RV-A16. Newly produced virus was harvested and the titer was determined by TCID_50_ assay. All tested compounds showed a substantially weaker effect if added after the virus entry. (D and E) BafA1, NH_4_Cl, niclosamide, and nigericin neutralize acidic intracellular compartments. HeLa-Ohio cells were treated with the indicated compounds for 1 h at 37°C at the maximum concentrations used for Fig. 1C to E. The presence of acidic endolysosomal compartments was quantified with LysoTracker Red DND-99. Panel D shows the granularity of the LysoTracker signal as quantified using CellProfiler (106). Panel E shows representative images of the LysoTracker signal quantified in panel D. DMSO, dimethyl sulfoxide.

RV-A16 was passaged on HeLa-Ohio cells in the presence of permissive concentrations of either bafilomycin A1 (BafA1) or NH_4_Cl, where the inhibitor concentrations were increased every 2 or 3 passages (Fig. 1A). After 10 passages, the resulting virus populations, termed P10, were tested for resistance against the inhibitors. The BafA1-selected P10 grew to slightly lower titers than the wild-type (wt) RV-A16 but remained largely unaffected by the addition of BafA1, unlike the wt A16, which was strongly attenuated by BafA1 (Fig. 1B). Sequencing of P10 viruses identified two mutations affecting the genomic region encoding VP1 to VP4. These mutations were reverse engineered into an RV-A16 infectious cDNA clone. We focused on two point mutations, G2274U from passage in NH_4_Cl and A2526G from BafA1 cultures. The former is located in the C-terminal domain of VP3 and alters cysteine 220 to phenylalanine (C220F), and the latter is in the N-terminal domain of VP1 and alters serine 66 to asparagine (S66N). Clonal virus populations with separate mutations were tested for susceptibility against BafA1, NH_4_Cl, and niclosamide (Fig. 1C to E). Both mutations conferred resistance not only to the original inhibitor but also to the second inhibitor, as well as a third inhibitor of endolysosomal acidification, niclosamide. Niclosamide is a weak acid, with a pK_a_ of 5.6, and acts as a protonophore equilibrating the luminal pH across lipid membranes (47, 48). The levels of inhibitor resistance of the two mutants were similar, although the VP3-C220F mutation conferred higher replication levels in the presence of BafA1 or niclosamide than the VP1-S66N mutation, while coxsackievirus B3 (CVB3), which infects independent of low endosomal pH and does not respond to niclosamide (49), was completely unaffected by the inhibitors (Fig. 1C and E). The double mutant carrying both the G2274U and A2526G mutations did not give rise to infectious virus (Fig. 2A). Remarkably, treatment with 70 mM NH_4_Cl led to a 1-log growth reduction of the pH-independent CVB3 (Fig. 1D). This could be interpreted as a stabilizing effect of ammonium ions on enteroviruses in general, as NH_4_Cl alone also stabilized wt RV-A16 (Fig. 2B). We further noted that the P10 populations replicated to lower titers than RV-A16 in the absence of endosomal acidification inhibitors (Fig. 1B), whereas the recombinant viruses raised from the infectious clone replicated to the same levels as RV-A16 (Fig. 1C to E). This effect was likely due to defective interfering particles upon virus passaging (50) or mutations in the genome not encoding structural proteins. To test whether BafA1, NH_4_Cl, and niclosamide affected viral entry, we added the inhibitors before or after virus entry, that is, 1 h before or after infection with RV-A16. All inhibitors had strong antiviral effects in the pretreatment regimen but not the postentry treatments (Fig. 2C). Importantly, all inhibitors were effective at neutralizing the endosomal pH (Fig. 2D and E). The data indicate that the G2274U and A2526G viruses arose in response to the acidification inhibitors interfering with entry rather than postentry events.

### The VP3-C220F and VP1-S66N mutations affect the interprotomeric interface.

**FIG 3:**
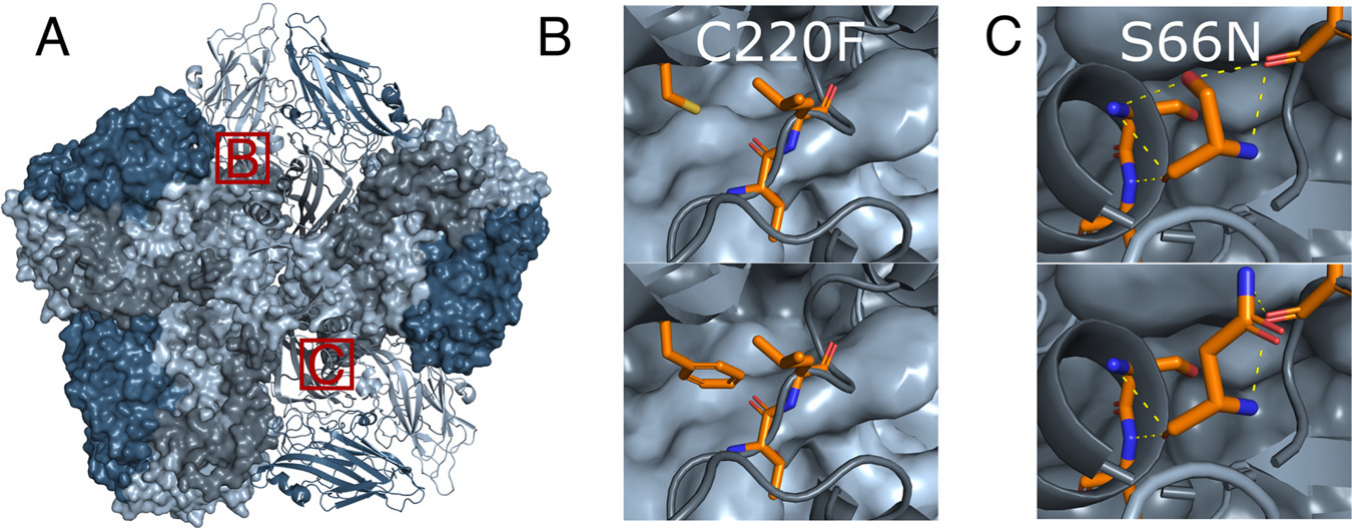
VP3-C220F and VP1-S66N mutations affect the interprotomeric interface. (A) Atomic model representing an inside view of one pentamer (PDB code 1AYM). Red boxes mark the zoomed-in areas in panels B and C. Neighboring protomers are displayed in surface view. Steel blue, VP2; light gray, VP3; dark gray, VP1; light blue, VP4. (B and C) Zoomed-in views showing the wt and the mutated amino acid residues in the upper and lower panels, respectively. Panel B depicts how the cysteine-to-phenylalanine change in VP3 at residue 220 leads to collisions with the neighboring VP1 lysine at position 61 (center of field of view). Panel C shows how the serine-to-asparagine change in VP1 at residue 66 leads to loss of a polar contact in the most probable rotamer.

The G2274U mutation (C220F) is close to the C terminus of VP3. In the mature particle, C220 is located at the interprotomeric interface (Fig. 3) (51). It is not exposed on the surface of the capsid and is unlikely directly affected by endosomal solutes. Yet, the cysteine residue in wt RV-A16 is much smaller than the bulky phenylalanine residue in the mutant virus. A phenylalanine at this position likely affects the interaction dynamics between the protomers and potentially the capsid stability. Based on the available structure of RV-A16 (PDB code 1AYM [51]), VP3-F220 in its most probable rotamer collides with the van der Waals radius of the peptide bond of the neighboring VP1-lysine 61 of the same protomer. The resulting displacement of this chain may have cascading effects on the interprotomeric contacts and modulate capsid dynamics. The amino acid change caused by the G2526A mutation (S66N) localizes to the N-terminal domain of VP1 and may lead to the loss of a polar contact of the serine side chain (Fig. 3C). Notably, the mutated amino acids are in close proximity to each other, highlighting the importance of the interprotomeric contact sites for virus resistance against inhibitors of endolysosomal acidification.

### The VP3-C220F and VP1-S66N mutations reduce capsid stability under near-physiological conditions.

**FIG 4:**
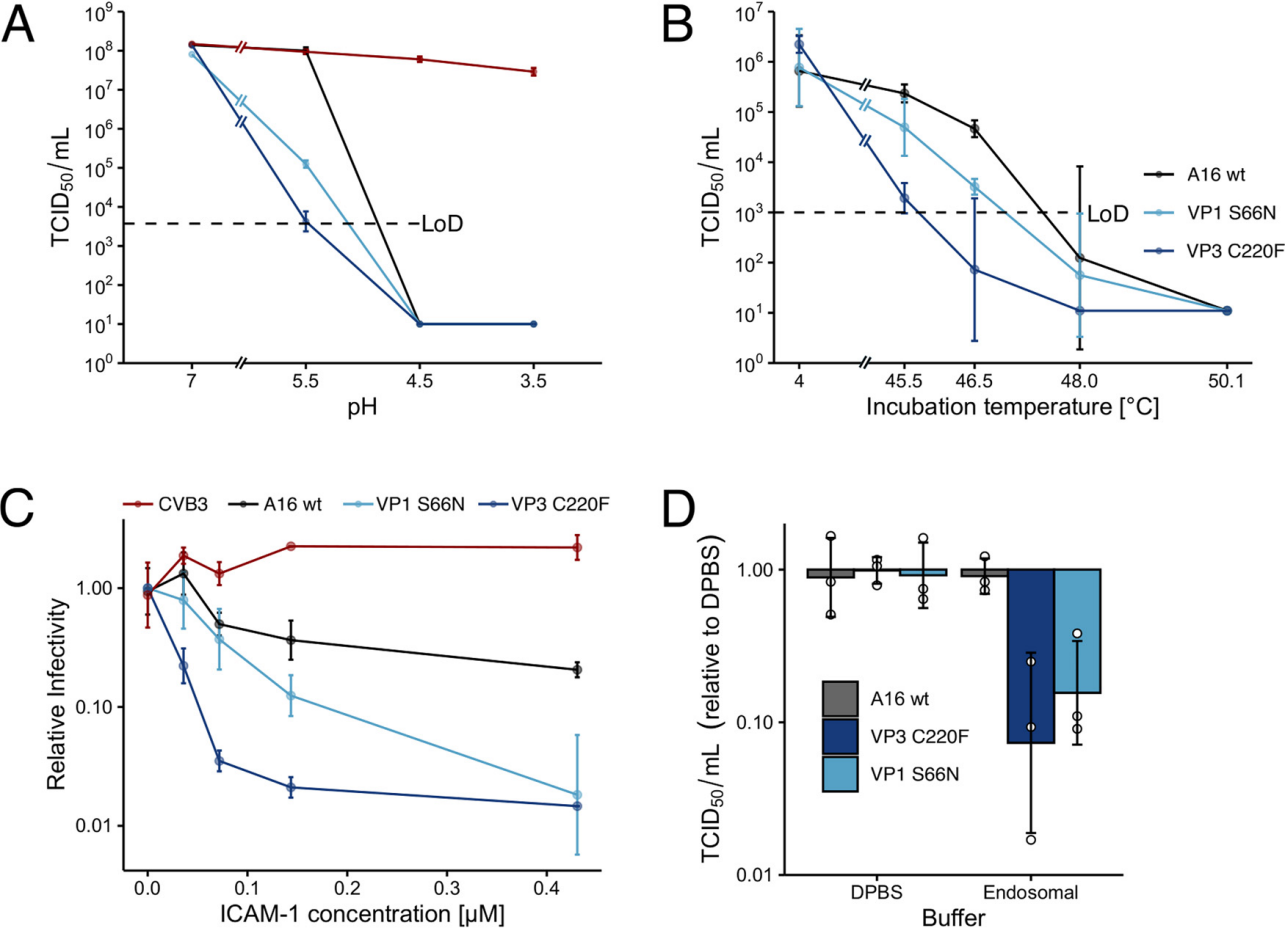
The VP3-C220F and VP1-S66N mutations reduce capsid stability. Graphs show the fractions of surviving virus as determined by endpoint titration. Mutants were more sensitive to all stress conditions, where the VP3-C220F (G2274U) mutant was the most sensitive one in all assays. (A) Resistance to low pH. Virus was incubated on dialysis filter membranes on PBS or acetate buffers under the indicated pH conditions. (B) Resistance to elevated temperatures. Virus was incubated at the indicated temperatures for 5 min. (C) Sensitivity to soluble ICAM-1 receptor domain. Indicated concentrations of soluble ICAM-1 were added to virus and incubated at 37°C for 30 min. (D) Virus sensitivity to endosomal-like ionic conditions. Virus was diluted in Dulbecco’s PBS (DPBS) or into a neutral pH endosomal-like buffer and incubated at 42°C for 5 min.

We next tested if adaptation to the absence of the low-pH uncoating cue affected the pH sensitivity of the virions. Wild-type and mutant virus particles were exposed to different pH conditions in a cell-free environment, and the remaining fraction of infectious virus was determined by titration (Fig. 4A). Both VP3-C220F (G2274U) and VP1-S66N (A2526G) were more pH labile than the wild type, while CVB3 remained unaffected by the treatments. To test whether this effect was observable under stress conditions other than low pH, we subjected the virus particles to heat stress (Fig. 4B). The VP3-C220F (G2274U) mutant had a drastically reduced resistance to temperature stress compared to that of wt RV-A16, while VP1-S66N (A2526G) was more destabilized by heat than the wt as well, albeit to a lesser extent than VP3-C220F.

We next tested the susceptibility of the viruses to soluble ICAM-1 (sICAM-1) receptor. ICAM-1 binding to RV-A16 primes the capsid for low-pH-mediated uncoating (20). The viruses were exposed to sICAM-1 at 37°C for 5 min. Strikingly, both mutants readily lost infectivity in a dose-dependent manner, while wt RV-A16 remained largely unaffected by sICAM-1 (Fig. 4C). Notably, the VP3-C220F mutant was more sensitive than VP1-S66N.

Following receptor binding, RV-A16 engages clathrin-mediated endocytosis and low pH to release its genome into the cytosol (20, 52). Since the ionic milieu of endosomes is distinct from the extracellular one (12, 53), we exposed the viruses to a buffer mimicking an endosomal ionic environment with intermediate concentrations of sodium, potassium, calcium, and chloride ions (20 mM NaCl, 30 mM KCl, and 0.2 mM CaCl_2_) in the presence of magnesium salt (0.5 mM MgCl_2_) at neutral pH or to Dulbecco’s phosphate-buffered saline (DPBS; 137.9 mM NaCl, 8.06 mM Na_2_HPO_4_, 1.47 mM KH_2_PO_4_ [pH 7.2]). Both mutants were readily inactivated in the endosomal buffer, unlike the parental virus and viruses incubated in Dulbecco’s phosphate-buffered saline containing high concentrations of sodium chloride (143 mM [Fig. 4D]). Again, the VP3-C220F mutant was more sensitive than VP1-S66N. Together, the data indicate that both interprotomeric mutations VP3-C220F and VP1-S66N predispose the viruses to physiological uncoating cues and reduce the overall resistance to acid and heat stress.

### Reproducible emergence of the capsid destabilizing mutations VP3-C220F and VP1-S66N.

**FIG 5:**
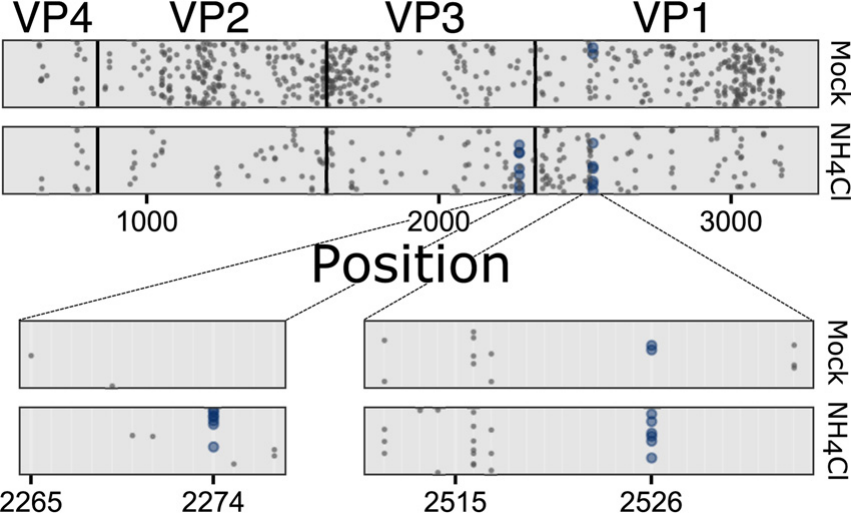
Analyses of frequency of RV-A16 mutations in presence or absence of NH_4_Cl reveal mutational hot spots at nucleotide positions 2274 and 2526. Ten separate lineages of wt RV-A16 were passaged 10 times in the presence or absence of NH_4_Cl, followed by massive parallel sequencing. Each data point represents a synonymous or nonsynonymous nucleotide variation with >1% frequency, where the mutations highlighted in blue are all nonsynonymous. Seven and six out of 10 lineages passaged on NH_4_Cl carried a variation at positions 2274 and 2526, compared to 0 and 2 in the control lines, respectively.

To explore the reproducibility of the mutations G2274U and A2526G, 10 separate lineages of wt RV-A16 were passaged with or without NH_4_Cl, followed by massive parallel RNA sequencing. Figure 5 shows all the variants with a frequency of greater than 1%. Spontaneous, synonymous mutations were abundant across the genome, regardless of whether NH_4_Cl was present. In the absence of NH_4_Cl, no variation was detected at nucleotide position 2274, while 7 of 10 lineages passaged in NH_4_Cl carried a point mutation at this position. Four of seven lineages carried the G2274U mutation encoding C220F, while the remaining three evolved a G2274A mutation giving rise to C220Y. Remarkably, both 220F and 220Y are amino acids with a bulky aromatic residue occupying considerably more space than 220C in wt RV-A16, in line with the interprotomeric mismatch described above. This finding was strengthened by the observation that the C2275G mutation gave rise to a nonsynonymous change of C220 to tryptophan (W), a bulky hydrophobic amino acid akin to phenylalanine or tyrosine (Fig. 5). This further strengthens the importance of the amino acid at position 220 at the interprotomeric interface in evolving viral resistance to acidification inhibitors. Many additional variants were also identified around position 2526 (Fig. 5). Six out of 10 NH_4_Cl lineages carried the A2526G (S66N) variant, compared to 2 out of 10 in the absence of NH_4_Cl. The other four lineages carried the wild-type A at position 2526. They may carry other mutations rendering them resistant to the effects of NH_4_Cl. Together, the data highlight the importance of a bulky amino acid at position 220 of VP3 and 66 of VP1 to destabilize the virion and render it independent of the acid cue for endosomal uncoating and infection.

**FIG 6:**
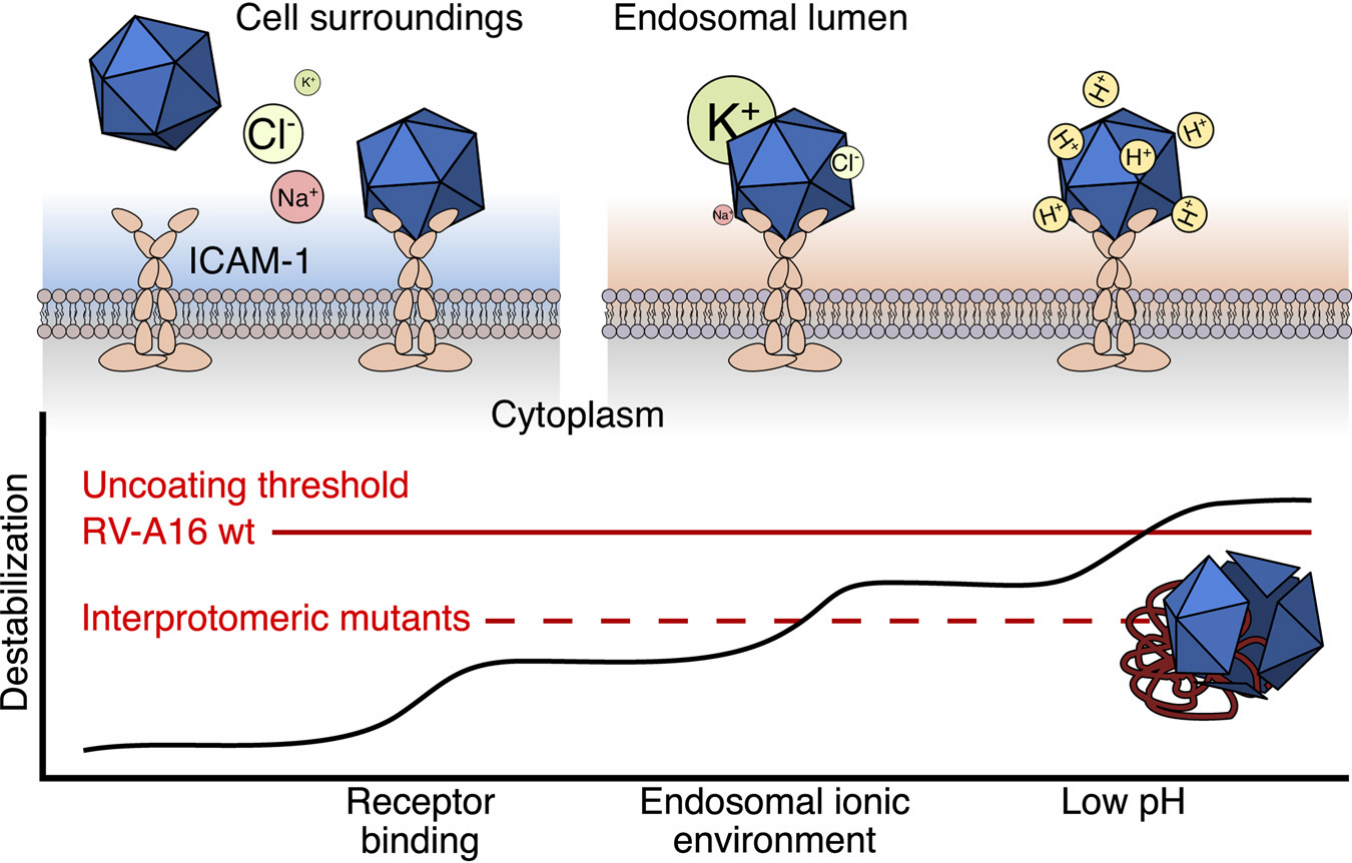
Differential susceptibility of the wt and the interprotomeric RV-A16 mutants VP3-C220F and VP1-S66N to cumulative destabilization by receptor binding, endosomal-like ionic conditions, and low pH. The data support a model in which the incoming virus particles are exposed to a series of uncoating cues in a stepwise manner, as originally established with human adenovirus (107). The initial cue occurs by ICAM-1 binding to the virion and destabilizes the particle by releasing the pocket factors. Upon endocytic uptake, the virion is exposed to a particular endosomal ionic environment with concentrations of Na^+^, K^+^, Cl^−^, Ca^2+^, and Mg^2+^ ions roughly intermediate between the extracellular medium and the cytosol, as well as progressively increasing proton concentration (53). While wt RV-A16 has a relatively high threshold for RNA uncoating, the RV-A16 mutants adapted to pH-neutral endosomes have a lower threshold. Mutant particles are readily inactivated by just one type of cue, such as ICAM-1 binding or endosomal-like ionic conditions, unlike wt RV-A16, which remains stable under these conditions. This indicates that the VP3-C220F and VP1-S66N mutants adapted to cells lacking low pH endosomes by reducing their capsid stability.

We suggest a model in which either of these two critical interprotomeric mutations in RV-A16 compensates for the lack of the low-pH uncoating cue and allows the virus to evade endosomal acidification inhibitors (Fig. 6). The incoming mutant particles still receive and respond to a series of uncoating cues, including ICAM-1 receptor binding and a low-sodium, low-chloride endosomal milieu, which allows for entry and uncoating in a stepwise manner (reviewed in references 46, 54, 55, and 56). Yet the mutant particles are less stable and less resistant to extracellular stress than the wild-type virus, which represents a severe fitness cost.

## DISCUSSION

Most human respiratory diseases have a viral etiology, yet we have insufficient countermeasures at hand against respiratory viruses. Reasons include the large viral diversity, emerging resistance against antiviral treatments, and the incomplete neutralization of different virus types by the immune system. For example, RVs evolve resistance against direct virus-targeting compounds, such as the purine RNA nucleotide prodrug ribavirin, the 3C protease inhibitor rupintrivir, and the uncoating inhibitor pleconaril (57–59). In this study, we explored the adaptation of RV-A16 to endosomal acidification inhibitors targeting the host, and we provide new insight into drug resistance, uncoating, and entry of RVs.

Low pH is an important cue for uncoating of many viruses, particularly those that are not exposed to acidic conditions of the gastrointestinal tract. For example, influenza A virus (IAV) hemagglutinin reacts to low pH by exposing the hydrophobic fusion peptide and inserts it into the limiting endosomal membrane, triggering viral fusion with the host membrane (60–62). The foot-and-mouth disease virus is probably the most pH-sensitive picornavirus, and it starts to dissociate into pentamers at slightly acidic pH, followed by genome uncoating and infection (63, 64). Likewise, the dissociation of pentamers from EV-18 facilitates directional RNA release, as shown by cryo-electron microscopy, arguing that pentamer release might represent a mechanism leading to infection (18).

By applying chemical evolutionary pressure, we identified novel RV-A16 variants which infected cells independent of low endosomal pH. Two different point mutants, VP3-C220F and VP1-S66N, were resistant against three distinct acidification inhibitors, the endolysosomotropic weak base NH_4_Cl (65), the vacuolar ATPase inhibitor BafA1 (66, 67), and niclosamide, a protonophore leading to pH equilibration across membranes (47). Notably, protonated niclosamide is more hydrophobic than the unprotonated form, which enhances membrane penetration and explains the rapid mode of action *in vivo* and in reconstituted lipid micelles (47). Neither VP3-C220F nor VP1-S66N is in close proximity to the ICAM-1 receptor binding site in the canyon region harboring the hydrophobic pocket, which provides a tuneable destabilization mechanism when the pocket factor is released upon receptor binding to the virion (68, 69). This makes it unlikely that the mutants affect the receptor binding affinity. Instead, we observed an increase of virion susceptibility to destabilization under various conditions, including low pH, temperature, and also binding of soluble receptor fragments. Both VP3-C220F and VP1-S66N located to the interface between protomers. The protomer interface is critical for stability and RNA uncoating of different picornaviruses, including CVB, enteroviruses, and the cardiovirus Saffold virus (70–72). The VP3-C220F and VP1-S66N mutations likely reduce overall RV-A16 capsid stability, and thereby provide the mechanism by which these viruses infect cells in the absence of low pH.

Our data suggest that receptor binding and the endosomal ionic environment at neutral pH in combination with the temperature of the upper respiratory tract sufficiently destabilize the mutant particles, such that genome release and infection occur. Structural compensation for the absence of a particular uncoating cue is in line with recent findings that the binding of sulfated glycosaminoglycans to EV-D68 induces changes in the virions and thereby renders the particles independent of the broadly required enterovirus entry factor phospholipase A2 (73, 74).

The ionic conditions in endolysosomes have been widely implicated in enhancing viral infections, in particular the Na^+^/K^+^-ATPase regulating endosomal pH (75), the Ca^2+^-selective two-pore channels (TPCs), and transient receptor potential mucolipins (TRPMLs) (reviewed in reference 76). Mutations in TRPMLs lead to enlarged endosomes and impaired endosomal lipid and protein trafficking, affecting endosomal maturation as well as reduced IAV, yellow fever virus, and Zika virus infections (77). The knockdown of TPC1 or TPC2, in turn, decreases the activity of the virion processing furin protease and reduces Middle East respiratory syndrome (MERS) coronavirus fusion with cellular membranes, as well as Ebolavirus infection (78, 79). Stepwise priming of IAV by high concentrations of K^+^ and low pH allows efficient virion uncoating and penetration of the RNA cores from the plasma membrane into the cell (80). Interestingly, bunyavirus infection also depends on high endosomal K^+^, which promotes virus progression through the endolysosomal system (81). Furthermore, echovirus 1 uncoating is facilitated by exposure of the particles to an endosomal buffer (12). All these data are in line with our observations that the exposure of RV-A16 to an endosomal-like, slightly hypotonic buffer with intermediate concentrations of Na^+^, K^+^, Cl^−^, Ca^2+^, and Mg^2+^ ions compared to the extracellular milieu and the cytosol provides the necessary cues together with ICAM-1 for the destabilization of the mutant capsids in the absence of low pH.

As most nonenteric animal viruses depend on endosomal low pH for uncoating or membrane fusion, inhibitors of endosomal acidification are attractive to target the host rather than the virus in antiviral therapy. This strategy may benefit from the notion that host targeting can affect a broad range of different viruses. It also benefits from the fact that viruses evolve slow resistance against host-targeting drugs and rather fast resistance against direct virus-targeting drugs (82).

Here, we have shown that RV-A16 has a limited set of possibilities to genetically adapt to and evade endosomal acidification inhibitors, as we reproducibly detected only two distinct point mutations rendering the virus resistant to the acidification inhibitors. Both mutations reduced the stability of the capsid. In a physiological setting, they may bear a high fitness cost, making it unlikely that the mutants will become prevalent in nature. Notably, the VP3-C220 residue is conserved across all RV-A types. All the available sequences of RV-B code for a lysine residue at this position, and in RV-C, they encode mostly a serine or alanine residue. None of the picornavirus genomes that we could access carries a phenylalanine at this position, unlike our acidification-independent RV-A16 mutants. Likewise, all RV-A types carry an alanine at position 66 of VP1 but never an asparagine, unlike our mutant RV-A16. The amino acid at position 66 is, however, less conserved in B and C types, but again, none of the available sequences carries an asparagine at position 66.

The high degree of amino acid conservation at VP3-220 and VP1-66 implies that these positions do not easily tolerate bulky or otherwise hydrophobic residues without severe fitness cost for the virus, at least *in vitro*, and possibly *in vivo*, based on the absence of these variants in nature. This may have clinical implications. Notably, FDA-approved endosomal acidification inhibitors such as niclosamide can potentially be repurposed against low-pH-dependent viruses. Niclosamide is an over-the-counter antihelminthic compound with a broad antiviral profile against acid-dependent viruses (47, 83, 84). Most recently, it has been considered for topical repurposing in the respiratory tract against COVID-19, caused by severe acute respiratory syndrome coronavirus 2 (SARS-CoV-2) (85, 86), and was effective against SARS-CoV-2-induced syncytium formation in cell cultures (87). Local application of niclosamide would stand in contrast to the systemic delivery of chloroquine and hydroxychloroquine, which failed in clinical trials against COVID-19 (88) and were reported to be noneffective against SARS-CoV-2 infections of cells expressing transmembrane protease serine subtype 2 (TMPRSS2) (89). Notably, the SARS-CoV-2 spike (S) protein undergoes three types of proteolytic cleavages. The first cleavage is mediated by furin at the S1/S2 site during S-protein biogenesis in acidic compartments of the secretory pathway and produces noncovalently linked S1-S2 heterodimers (90). Importantly, the S1/S2 cleavage site is maintained in human evolution of SARS-CoV-2 variants, possibly providing a fitness advantage for virus, as suggested by reduced transmissibility of furin cleavage-defective SARS-CoV-2 in hamster models (91). The second S-protein cleavage occurs by TMPRSS2 at the S2′ site of the membrane-anchored S2 on the cell surface, and the third cleavage occurs by cathepsin L during virus entry in acidic endosomes (reviewed in reference 92). While both angiotensin-converting enzyme 2 (ACE2) and neuropilin 1 (NRP1) receptors bind to the S1 fragment (93, 94), furin cleavage and NRP1 binding to S1 enhance accessibility of S2 to TMPRSS2 and activation of membrane fusion at the cell surface (95). In the absence of TMPRSS2, however, SARS-CoV-2 enters cells by ACE2-mediated endocytosis, undergoes proteolytic activation by cathepsin L in low-pH-containing endosomes, and fuses its membrane with a limiting endosomal membrane for infection (96). We surmise that in the absence of low-pH compartments, SARS-CoV-2 activation by TMPRSS2 is less effective, thus forcing the virus to enter by an endocytic route, albeit with suboptimal cathepsin L activation and hence lower infectivity. SARS-CoV-2 adaptation to cells lacking low-pH intracellular compartments may thus cost viral fitness. For example, virus would have to compensate for multiple aspartic acid residues acting as pH switches in the S-protein trimers undergoing conformational transitions at low pH and coordinating movements of interprotomer domains (97). Accordingly, such mutant viruses may exhibit fitness cost, perhaps akin to our RV-A16 mutants.

In conclusion, our study shows that the inhibition of endosomal acidification selects for two distinct RV-A16 escape mutants with impaired capsid stability. Such escape mutants likely suffer from a severe fitness disadvantage under physiological conditions. The data encourage the development and application of endosomal acidification inhibitors for a broad treatment of RVs and the common cold, without *a priori* concerns of raising unpredictable gain-of-function mutants.

## MATERIALS AND METHODS

### Chemicals, antibodies, cell lines, and viruses.

BafA1 (B1793), NH_4_Cl (A4514), nigericin (N7143), and niclosamide (N3510) were obtained from Sigma-Aldrich (St. Louis, MO). The efficacy of the inhibitors to neutralize endolysosomal pH was demonstrated with the LysoTracker DND-99 assay (Life Technologies), as described previously (98). The original infectious cDNA clone of RV-A16 (pR16.11) was a gift from W. M. Lee (Department of Pediatrics, School of Medicine and Public Health, University of Wisconsin, WI) (99). CVB3 strain Nancy was used as described previously (100). HeLa-Ohio (ECACC 84121901) cells were obtained from L. Kaiser, Central Laboratory of Virology, University Hospital Geneva, Switzerland. Cells were cultured in Dulbecco’s modified Eagle’s medium (DMEM; D6429; Sigma-Aldrich) supplemented with 10% fetal bovine serum (FBS; 10270; Invitrogen, Carlsbad, CA) and 1% nonessential amino acids (NEAA; M7145; Sigma-Aldrich), washed in PBS, and detached with trypsin-EDTA (C-41020; Sigma-Aldrich). Cells were kept at 5% CO_2_, 95% humidity, and 37°C.

### BafA1- and NH_4_Cl-resistant viruses.

HeLa-Ohio cells were seeded in 6-well plates and incubated overnight at 37°C. Cells were initially infected at a multiplicity of infection (MOI) of 0.01 in the presence of either 0.5 nM BafA1 or 1 mM NH_4_Cl. Once 90% of cells showed cytopathic effect (CPE), supernatant was harvested and cleared by centrifugation at 10,000 × *g* for 5 min at room temperature (RT). Thirty microliters of the clear cell lysate was used to initiate the next passage. This procedure was repeated 10 times. Endosomal acidification inhibitor concentrations were increased after every second or third passage. In the repeated-passaging experiment, HeLa-Ohio cells were seeded in 12-well plates and incubated overnight at 37°C. Cells were infected at an MOI of 0.01 in the presence of 3 mM NH_4_Cl, and the clear cell lysate volume to initiate the next passage was 10 μL. Otherwise, the conditions were kept exactly as outlined above. Ten populations were passaged in parallel per condition (with or without NH_4_Cl).

### RNA extraction by TRIzol.

Virus-containing supernatants were treated with RNase A/T1 mix (EN0551; Thermo Fisher Scientific, Waltham, MA) at 37°C for 30 min. Subsequently, supernatants were mixed 1:1 with TRIzol and centrifuged at 12,000 × *g* for 10 min at 4°C. The aqueous phase was collected and the RNA was precipitated with isopropanol (59309; Sigma-Aldrich, St. Louis, MO).

### RNA extraction by spin column purification.

Clear cell lysates were thawed, 200 μL of each sample was treated with RNase A/T1 mix (EN0551; Thermo Fisher Scientific, Waltham, MA) for 1 h at 37°C, and subsequently RNA was extracted with the High Pure RNA isolation kit (11828665001; Roche, Basel, Switzerland) according to the manufacturer’s instructions.

### Massive parallel sequencing of viral RNA.

RNA extracts were treated with TURBO DNase (AM2238; Thermo Fisher Scientific, Waltham, MA) and purified using RNAClean XP beads (A63987; Beckman Coulter, Brea, CA). Next-generation sequencing (NGS) libraries were prepared using the Trio RNA-Seq library preparation kit (NuGen). Sequencing was performed on an Illumina NextSeq500 (Illumina, San Diego, CA) using single-end 150-bp reads.

### Sequencing data analysis.

Reads were first trimmed for low-quality nucleotides and adapters using BBDuk from the BBTools suite (101). Trimmed reads were aligned using BBMap and BWA (102). Variants were called using LoFreq (103) and output generated with VCFtools (104).

### Reverse engineering and generation of clonal mutant viruses.

Point mutations were introduced to pR16.11 with the Q5 site-directed mutagenesis kit (E0554S; New England BioLabs, Ipswich, MA) or the KOD polymerase (71085; Sigma-Aldrich, St. Louis, MO). Infectious clones were *in vitro* transcribed with the HiScribe T7 high-yield RNA synthesis kit (E2040S; New England BioLabs). RNA was collected by phenol-chloroform extraction (77617; Sigma-Aldrich) and transfected into HeLa-Ohio cells with the TransIT-mRNA transfection kit (MIR 2225; Mirus Bio, Madison, WI) according to the manufacturer’s conditions. Cells were harvested after 72 h and subjected to 2 freeze-thaw cycles, and the lysates were cleared by centrifugation at 10,000 × *g* at RT.

### Virus replication assays.

Cells were pretreated with the desired concentrations of BafA1, NH_4_Cl, or niclosamide at 37°C for 30 min. The samples were then infected at an MOI of 0.01 at 33.5°C for 30 min. The virus inoculum was removed, cells were washed with PBS, and the medium containing inhibitors was replenished. Virus was harvested by freeze-thaw cycling of the cells after 8 or 16 h. Newly generated virus was quantified by 50% tissue culture infective dose (TCID_50_) assays using the Reed-Muench method (105). The limit of detection was reached when the lowest tested virus concentration gave rise to cytopathic effect in 50% of the tested wells.

### Virus inactivation by pH, temperature, and soluble ICAM-1 receptor.

Viruses were brought to the same titer by dilution in DMEM plus 2% fetal calf serum (FCS). For the temperature sensitivity, samples were then exposed to heat in a thermocycler for 30 min and then cooled down to 4°C until titration. For pH sensitivity, viruses were dialyzed against isotonic buffers (150 mM NaCl, 25 mM NaOAc for pH of ≤5.5, or PBS) under the desired pH conditions at 4°C for 16 h using dialysis filter membranes (VSWP02500; Millipore, Burlington, MA). Virus was collected, incubated at 37°C for 20 min, and brought back to pH 7.4. The titer of the surviving fraction was determined by TCID_50_ assay. For the ICAM-1 sensitivity assay, viruses were brought to the same concentration and were then diluted 1:1 in solution containing soluble ICAM-1 (BMS313; Invitrogen, Carlsbad, CA) or PBS only. Subsequently, samples were incubated at 37°C for 15 min and titers were determined for infectivity. To determine the sensitivity to the endosomal ionic environment, viruses were brought to the same titer and diluted 1:75 in DPBS (with Ca^2+^ and Mg^2+^; pH 7.2) or endosomal buffer (20 mM NaCl, 30 mM KCl, 0.5 mM MgCl_2_, 0.2 mM CaCl_2_. 8.06 mM Na_2_HPO_4_, 1.47 mM KH_2_PO_4_ [pH 7.2]).

### Data availability.

The full sequencing data used and interpreted in this article are available at https://data.mendeley.com/datasets/bbhw6ff2s5/1.
